# Gut Microbiota Predict *Enterococcus* Expansion but Not Vancomycin-Resistant *Enterococcus* Acquisition

**DOI:** 10.1128/mSphere.00537-20

**Published:** 2020-11-18

**Authors:** Rishi Chanderraj, Christopher A. Brown, Kevin Hinkle, Nicole Falkowski, Piyush Ranjan, Robert P. Dickson, Robert J. Woods

**Affiliations:** a Division of Infectious Diseases, Department of Internal Medicine, University of Michigan Medical School, Ann Arbor, Michigan, USA; b Division of Pulmonary and Critical Care Medicine, Department of Internal Medicine, University of Michigan Medical School, Ann Arbor, Michigan, USA; c Department of Microbiology and Immunology, University of Michigan Medical School, Ann Arbor, Michigan, USA; d Computational Medicine and Bioinformatics, University of Michigan Medical School, Ann Arbor, Michigan, USA; University of Wisconsin—Madison

**Keywords:** microbiome, vancomycin-resistant *Enterococcus*, colonization resistance, hospital-acquired infection

## Abstract

Vancomycin-resistant *Enterococcus* (VRE) is a leading cause of hospital-acquired infections and continues to spread despite widespread implementation of pathogen-targeted control guidelines. Commensal gut microbiota provide colonization resistance to VRE, but the role of gut microbiota in VRE acquisition in at-risk patients is unknown. To address this gap in our understanding, we performed a case-control study of gut microbiota in hospitalized patients who did (cases) and did not (controls) acquire VRE. We matched case subjects to control subjects by known risk factors and “time at risk,” defined as the time elapsed between admission until positive VRE screen. We characterized gut bacterial communities using 16S rRNA gene amplicon sequencing of rectal swab specimens. We analyzed 236 samples from 59 matched case-control pairs. At baseline, case and control subjects did not differ in gut microbiota when measured by community diversity (*P* = 0.33) or composition (*P* = 0.30). After hospitalization, gut communities of cases and controls differed only in the abundance of the *Enterococcus*-containing operational taxonomic unit (OTU), with the gut microbiota of case subjects having more of this OTU than time-matched control subjects (*P* = 0.01). Otherwise, case and control communities after the time at risk did not differ in diversity (*P* = 0.33) or community structure (*P* = 0.12). Among patients who became VRE colonized, those having the *Blautia*-containing OTU on admission had lower *Enterococcus* relative abundance once colonized (*P* = 0.004). Our results demonstrate that the 16S profile of the gut microbiome does not predict VRE acquisition in hospitalized patients, likely due to rapid and profound microbiota change. The gut microbiome does not predict VRE acquisition, but it may be associated with *Enterococcus* expansion, suggesting that these should be considered two distinct processes.

**IMPORTANCE** The Centers for Disease Control and Prevention estimates that VRE causes an estimated 54,000 infections and 539 million dollars in attributable health care costs annually. Despite improvements in hand washing, environmental cleaning, and antibiotic use, VRE is still prevalent in many hospitals. There is a pressing need to better understand the processes by which patients acquire VRE. Multiple lines of evidence suggest that intestinal microbiota may help some patients resist VRE acquisition. In this large case-control study, we compared the 16S profile of intestinal microbiota on admission in patients that did and did not subsequently acquire VRE. The 16S profile did not predict subsequent VRE acquisition, in part due to rapid and dramatic change in the gut microbiome following hospitalization. However, *Blautia* spp. present on admission predicted decreased *Enterococcus* abundance after VRE acquisition, and *Lactobacillus* spp. present on admission predicted *Enterococcus* dominance after VRE acquisition. Thus, VRE acquisition and domination may be distinct processes.

## Abstract (precis)

The Centers for Disease Control and Prevention estimates that VRE causes an estimated 54,000 infections and 539 million dollars in attributable health care costs annually. Despite improvements in hand washing, environmental cleaning, and antibiotic use, VRE is still prevalent in many hospitals. There is a pressing need to better understand the processes by which patients acquire VRE. Multiple lines of evidence suggest that intestinal microbiota may help some patients resist VRE acquisition. In this large case-control study, we compared the 16S profile of intestinal microbiota on admission in patients that did and did not subsequently acquire VRE. The 16S profile did not predict subsequent VRE acquisition, in part due to rapid and dramatic change in the gut microbiome following hospitalization. However, *Blautia* spp. present on admission predicted decreased *Enterococcus* abundance after VRE acquisition, and *Lactobacillus* spp. present on admission predicted *Enterococcus* dominance after VRE acquisition. Thus, VRE acquisition and domination may be distinct processes.

## INTRODUCTION

Vancomycin-resistant *Enterococcus* (VRE) species are highly antibiotic-resistant bacteria, are a leading cause of health care-associated infections, and are classified as a serious public health threat by the Centers for Disease Control and Prevention (1, 2). Colonization with VRE precedes infection (3, 4), and molecular epidemiologic analyses show patient-to-patient hospital transmission is the primary means of spread (5). Preventing transmission between hospitalized patients is a significant challenge, and despite the widespread application of pathogen-targeted control measures (6), VRE remains prevalent in many hospitals (1, 2).

Both indirect human evidence and animal experimentation demonstrate that gut microbiota prevent VRE colonization when a patient is exposed, a phenomenon termed “colonization resistance” (7–9). Colonization resistance may entail competition for resources, secretion of bactericidal factors (10, 11), and indirect stimulation of host immune defense mechanisms that target VRE (12, 13). Though colonization resistance plays a crucial role in suppressing VRE expansion and preventing VRE infection (14, 15), to date, no study has evaluated whether variation in intestinal microbiota can explain variation in VRE acquisition among at-risk patients.

To address this gap in our understanding of VRE transmission, we investigated whether the gut microbiome of at-risk patients predicts VRE colonization in a hospitalized patient population. We hypothesized that if the gut microbiome can confer colonization resistance for VRE acquisition, variation in baseline microbiota would explain variation in patient susceptibility to VRE acquisition. To test this hypothesis, we designed a case-control study using 16S rRNA gene amplicon sequencing of rectal swabs acquired from hospitalized patients.

## RESULTS

### Study population and medication exposures.

We studied gut microbiome communities in 236 rectal swab samples from 59 matched pairs of case and control subjects (Table 1). Cases and controls did not differ in demographics (age, sex, ethnicity) or in the relative frequency of common comorbidities (e.g., immunosuppression, malignancy, or gastrointestinal disease). Antibiotic use was widespread among all subjects and was nearly equal across groups (Table 2). Vancomycin, cefepime, metronidazole, and piperacillin-tazobactam were the most commonly used antibiotics in the study population. Cases and controls did not differ significantly in their exposure to any specific antibiotics prior to initial sampling. More cases received proton pump inhibitors prior to initial sampling (*P* = 0.04). During time at risk (between initial and subsequent sampling), case and controls did not differ in their exposure to antibiotics or proton pump inhibitors.

**TABLE 1:** Demographics and comorbidities of matched cohorts^*a*^

Demographic or clinical characteristic^*b*^	No. of individuals (proportion) with characteristic or value specified		*P* value
	Controls (*n* = 59)	Cases (*n* = 59)	
Demographics			
Age (mean ± SE)	57.19 ± 1.62	60.2 ± 1.95	0.23
Female	23 (0.39)	22 (0.38)	0.56
Nonwhite race	9 (0.15)	9 (0.15)	0.28
Diagnoses and comorbidities			
C. difficile infection	4 (0.07)	11 (0.18)	0.07
Leukemia	21 (0.36)	17 (0.29)	0.38
Lymphoma	12 (0.21)	10 (0.17)	0.49
Bone marrow transplant	15 (0.25)	14 (0.24)	0.64
Solid organ malignancy	35 (0.6)	40 (0.67)	0.33
Metastatic malignancy	29 (0.49)	35 (0.59)	0.10
Diabetes	27 (0.46)	23 (0.39)	0.59
Coronary artery disease	6 (0.11)	10 (0.17)	0.72
Congestive heart failure	19 (0.32)	18 (0.3)	0.60
COPD	21 (0.35)	36 (0.61)	0.02
Peripheral vascular disease	6 (0.1)	2 (0.03)	0.31
End-stage renal disease	18 (0.31)	26 (0.44)	0.07
Connective tissue disease	1 (0.01)	4 (0.06)	0.35
Peptic ulcer disease	9 (0.15)	6 (0.11)	0.50
Cirrhosis	2 (0.04)	9 (0.15)	0.06
Cerebrovascular disease	12 (0.21)	17 (0.29)	0.54
Hemiplegia	4 (0.06)	12 (0.2)	0.07
Dementia	1 (0.01)	3 (0.05)	0.34
Charlson score (mean ± SE)	3.71 ± 0.22	4.45 ± 0.25	0.05

a Cases and controls were matched by “time at risk” and unit or ward.

b C. difficile, Clostridium difficile; COPD, chronic obstructive pulmonary disease.

**TABLE 2:** Medication exposure of matched cohorts

Sampling time and medication	Prevalence of exposure^*a*^		*P* value	Duration of exposure^*b*^		*P* value
	Controls	Cases		Controls	Cases	
Prior to admission swab						
Antibiotics						
Any antibiotics	29 (0.49)	40 (0.68)	0.05	1.72 ± 0.62	2.32 ± 0.47	0.44
Vancomycin	14 (0.24)	21 (0.36)	0.17	0.52 ± 0.3	0.36 ± 0.06	0.61
Metronidazole	8 (0.14)	14 (0.24)	0.17	0.14 ± 0.04	0.4 ± 0.16	0.16
Piperacillin-tazobactam	8 (0.14)	12 (0.2)	0.29	0.14 ± 0.04	0.21 ± 0.06	0.25
Cefepime	7 (0.12)	11 (0.19)	0.32	0.4 ± 0.3	0.32 ± 0.12	0.81
Proton pump inhibitors	9 (0.15)	19 (0.32)	0.04	0.2 ± 0.07	0.61 ± 0.19	0.07
Between admission and “time at risk” swab						
Antibiotics						
Any antibiotics	56 (0.95)	52 (0.88)	0.18	21.66 ± 4.98	22.36 ± 3.66	0.82
Vancomycin	37 (0.63)	39 (0.66)	0.66	3.55 ± 0.82	3.33 ± 0.82	0.73
Metronidazole	20 (0.34)	24 (0.41)	0.43	2.01 ± 0.72	2.29 ± 0.6	0.75
Piperacillin-tazobactam	26 (0.44)	25 (0.42)	0.83	3.66 ± 1.13	2.8 ± 0.64	0.42
Cefepime	21 (0.36)	24 (0.41)	0.56	2.92 ± 1.01	3.1 ± 0.82	0.85
Proton pump inhibitors	33 (0.56)	39 (0.66)	0.23	5.15 ± 1.48	6.75 ± 1.32	0.13

a Prevalence values are reported as number of case or control individuals (proportion).

b Duration values are reported as numbers of days of therapy ± standard deviation (SD).

### Admission gut microbiota do not predict VRE acquisition.

**FIG 1:**
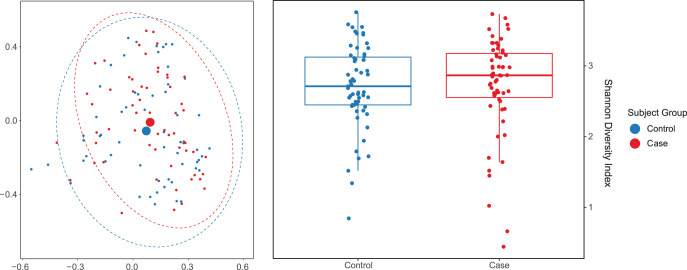
In hospitalized patients, admission gut microbiota do not predict subsequent VRE acquisition. We used 16S rRNA sequencing to characterize gut bacterial communities in 118 hospitalized patients who tested negative for VRE at admission. We compared admission gut microbiota across 59 matched cases (patients who acquired VRE) and controls (patients who did not acquire VRE). (Left) Visualization of admission gut microbial communities using principal-component analysis. The admission gut communities of cases and controls did not differ in their community composition, either visually or via permutation testing (*P* = 0.3 by PERMANOVA). (Right) Comparison of average species diversity as measured by Shannon diversity index in admission gut communities. The admission gut communities of cases and controls did not differ in their community Shannon diversity index (*P* = 0.96 by conditional logistic regression).

We first compared baseline microbiota across patients who did (cases) and did not (controls) subsequently acquire VRE. Baseline gut communities of cases and controls did not differ in their community composition, determined either via permutation testing (*P* = 0.30 by permutational multivariate analysis of variance [PERMANOVA]) or via visualization (principal-component analysis; Fig. 1, left). Similarly, baseline gut communities of cases and controls did not differ in their species diversity as measured by the Shannon diversity index (mean of 2.72 ± 0.90 for controls, mean of 2.71 ± 0.76 for cases, *P* = 0.96 for all matched case-control pairs) (Fig. 1, right). We noted that *Enterococcus* (OTU0004) was among the top 10 operational taxonomic units (OTUs) identified in both cases and controls at the time of admission. Despite the high abundance of the genus *Enterococcus* on admission, none of these swabs had vancomycin-resistant *Enterococcus* at the time of admission when evaluated with VRESelect chromogenic medium. *Enterococcus* (OTU0002) colonization did not imply VRE colonization, as OTU0004 captured both resistant and sensitive strains. We concluded that the gut microbiota, as represented by the 16S profile of these samples of hospitalized patients, do not predict subsequent VRE acquisition.

### At the time of VRE detection, the gut communities of cases and controls differ only in the abundance of *Enterococcus*.

**FIG 2:**
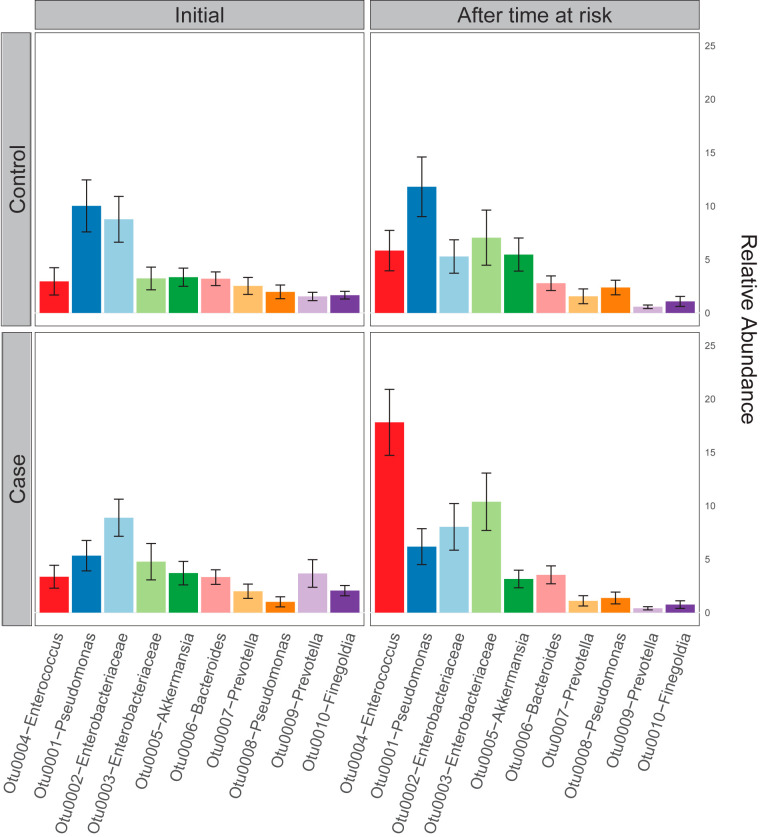
After the time at risk, the gut microbiota of cases and controls differ primarily in their relative abundance of *Enterococcus*. The 10 most abundant bacterial taxa are shown in controls (top) and cases (bottom), at the time of admission (left), and following matched time at risk (right). Cases and controls did not differ in their admission microbiota (left). After the time at risk, the gut microbiota of cases were enriched with *Enterococcus* relative to control (*P* < 0.01, mvabund), but otherwise did not differ in their community composition (*P* > 0.05 for all other taxa, mvabund).

**FIG 3:**
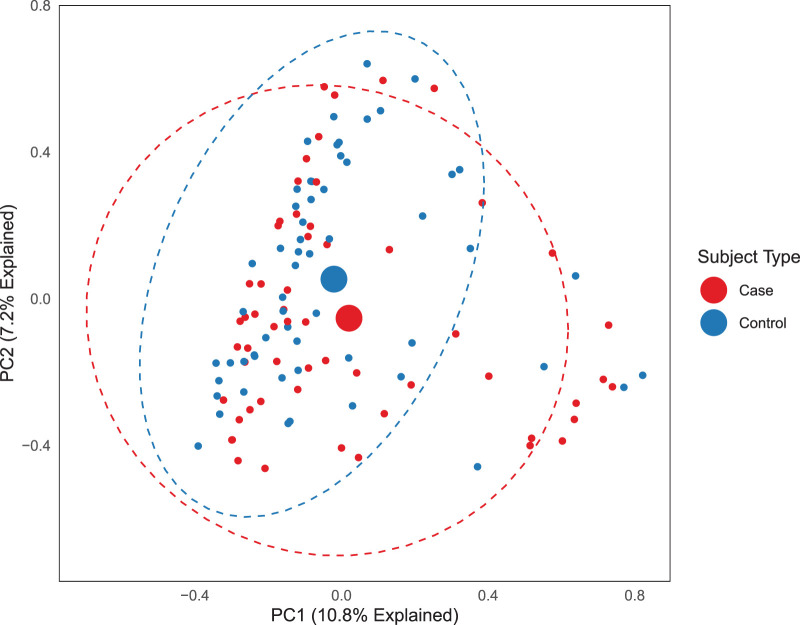
With the exception of *Enterococcus*, gut communities of VRE-infected and uninfected patients do not differ. When we excluded *Enterococcus* OTU enriched in VRE-infected patients, we found no remaining difference in bacterial community composition, either visually (principal-component analysis) or via permutation testing (*P* = 0.12 by PERMANOVA).

We next compared gut communities across matched cases and controls after time at risk: after cases had been colonized and time-matched controls had not. After time at risk, gut microbiota did differ across cases and controls (*P* < 0.001 by PERMANOVA), though Shannon diversity index did not (mean of 2.38 ± 0.11 for controls, mean of 2.22 ± 0.12 for cases, *P* = 0.33 for all matched case-control pairs). The difference in gut microbiota was driven by the increased relative abundance of a single OTU, the *Enterococcus*-classified taxonomic group (OTU0004), which was greater in cases than controls (*P* = 0.01 via *mvabund*, *P* < 0.001 via random forest) (Fig. 2). When this *Enterococcus* OTU was excluded from the analysis, we found no significant difference in communities across cases and controls (*P* = 0.12 by PERMANOVA) (Fig. 3). We thus concluded that at the time of VRE acquisition, the gut microbiota of VRE-infected and uninfected patients differ only in the relative abundance of *Enterococcus* and do not consistently differ in their non-*Enterococcus* microbiota.

### Gut microbiota change rapidly and profoundly in hospitalized patients.

**FIG 4:**
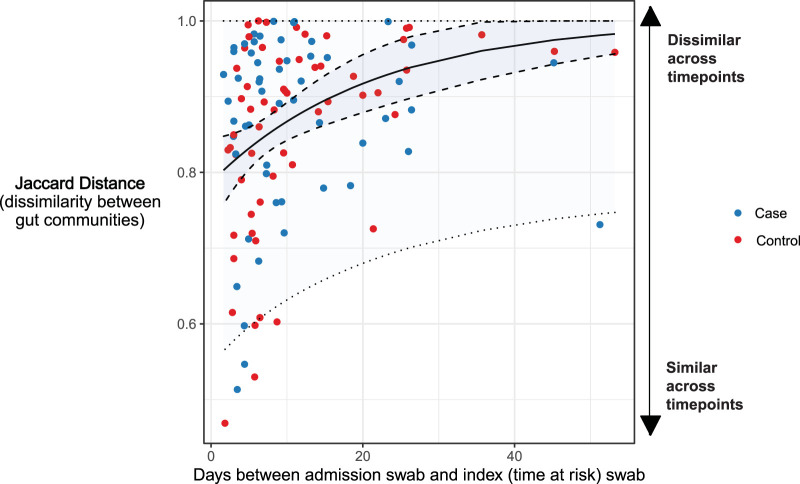
Rapid and dramatic change in gut microbiota among hospitalized patients. We calculated the dissimilarity between admission and subsequent (index, time at risk) gut communities in both cohorts with Jaccard distance. Both cases and controls exhibited rapid changes in gut communities during hospitalization, with Jaccard distance changing at an exponential rate. Cases and controls did not differ from each other in temporal disruption of gut microbiota. Dashed lines in the figure represent the 95% confidence interval for predicted mean Jaccard distance (inner ribbon) and predicted Jaccard distance for an individual subject (outer ribbon).

Given the lack of differentiation of gut communities across cases and controls at admission and at the time of VRE colonization, we then asked whether the temporal change in gut microbiota could distinguish the two groups. We did this by calculating the relative dissimilarity of admission and index (time-at-risk) communities for each subject using Jaccard distance, a metric of dissimilarity between gut microbial communities measured on a scale of 0 (complete similarity) to 1 (complete dissimilarity) (Fig. 4). The gut communities of both groups underwent a rapid, profound change in composition. Within several days of admission, gut communities of both cases and controls bore little similarity to the communities detected at the time of admission. The size of the change in gut communities did not differ across cohorts (0.87 ± 0.02 for cases, 0.86 ± 0.02 controls, *P* = 0.84). Cases and controls also had similar decreases in Shannon diversity (−0.48 ± 0.10 for cases, −0.40 ± 0.08 for controls, *P* = 0.93 for all matched case-control pairs). We found that Jaccard distance was significantly correlated with time (Spearman’s rank correlation coefficient ρ = 0.32, *P* = 0.0006) and determined that a negative exponential model best fit the data, with gut microbiota approaching complete dissimilarity at an exponential rate of 0.47 × *e*^−0.47t+32^ (*t* representing the time between swabs). We found no significant difference in the rate of change between the two groups. We noted that the predicted mean Jaccard distance for two rectal swab samples taken on the same day (*t* = 0) was 0.79 ± 0.058, implying a substantial amount of variation in community structure within the same day of admission.

### Gut microbiota on admission are associated with *Enterococcus* expansion.

**FIG 5:**
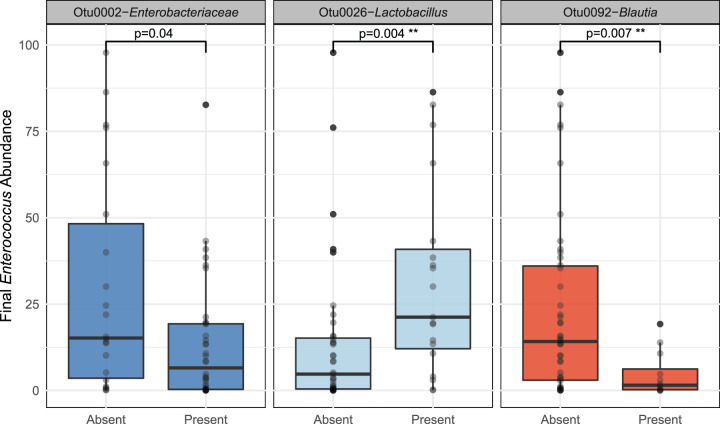
Presence of *Blautia* species on admission is predictive of decreased *Enterococcus* abundance at the time of VRE acquisition. A random forest regression model identified seven OTUs present on admission that predicted subsequent relative abundance of *Enterococcus* spp. Of these, the presence of *Enterobacteriaceae* spp., *Lactobacillus* spp., and *Blautia* spp. were significant predictors of the final relative abundance of *Enterococcus* spp.. Only *Lactobacillus* spp. and *Blautia* spp. remained significant after correcting for multiple testing. Significance was determined using the Mann-Whitney U test controlled for multiple comparisons.

Finding no difference in the community composition, diversity, or temporal rate of change across patients who did (cases) and did not (controls) acquire VRE during their hospitalization, we asked whether gut microbiota on admission could predict the relative abundance of *Enterococcus* in VRE-colonized patients. We built a random forest regression model to identify taxa present on admission that were predictive of final *Enterococcus* relative abundance. In cases, only *Blautia* and *Lactobacillus* were significant after correcting for multiple testing and feature importance bias (Fig. 5; see Tables S1 and S2 and Fig. S2 in the supplemental material). In cases, *Blautia* spp. (OTU 0092) on admission was predictive of decreased *Enterococcus* (−10.3% relative-abundance-adjusted *P* [relative abundance *P*] = 0.004 by Mann-Whitney U test), and *Lactobacillus* spp. (OTU 0026) was predictive with an increased abundance of *Enterococcus* after the time at risk (+12.5% relative abundance *P* = 0.007 by Mann-Whitney U test). A random forest regression model applied to the control population identified the same *Lactobacillus* and *Blautia* taxa as predictive of *Enterococcus* abundance after the time at risk (–3.7% relative abundance *P* = 2.4 × 10^−8^ and +3.6% relative abundance for *Lactobacillus P* = 0.003 by Mann-Whitney U test). In controls, *Lactobacillus* and *Blautia* were not the only predictive taxa, as *Phascolarctobacterium*, *Prevotella*, and *Bifidiobacterium* were also predictive of decreased *Enterococcus* abundance. While more taxa were predictive of final *Enterococcus* abundance in controls, the effect size of these taxa was smaller, as controls had a lower abundance of *Enterococcus* after the time at risk (Table S2). Thus, we found that the presence of specific anaerobes previously implicated in *Enterococcus* colonization resistance (8, 15, 16) is predictive of decreased *Enterococcus* abundance in both VRE-colonized and uncolonized patients. These findings suggest that VRE acquisition and *Enterococcus* expansion are two distinct processes with different risk factors and pathophysiology.

10.1128/mSphere.00537-20.1 TABLE S1 Most important taxa identified by random forest regression model in cases. We built a random forest regression model to identify taxa present on admission that were predictive of final *Enterococcus* relative abundance. In cases, only *Blautia* and *Lactobacillus* were significant after correcting for multiple testing and feature importance bias. Download Table S1, DOCX file, 0.01 MB. Copyright © 2020 Chanderraj et al. 2020 Chanderraj et al. This content is distributed under the terms of the Creative Commons Attribution 4.0 International license.

10.1128/mSphere.00537-20.2 TABLE S2 Most important taxa identified by random forest regression model in cases. We built a random forest regression model to identify taxa present on admission that were predictive of final *Enterococcus* relative abundance. In cases, only *Blautia* and *Lactobacillus* were significant after correcting for multiple testing and feature importance bias. Download Table S2, DOCX file, 0.01 MB. Copyright © 2020 Chanderraj et al. 2020 Chanderraj et al. This content is distributed under the terms of the Creative Commons Attribution 4.0 International license.

## DISCUSSION

In this study, gut microbiota did not predict VRE acquisition in hospitalized patients. Secondary analysis identified individual members of the gut microbiota that do predict *Enterococcus* abundance at the time of VRE acquisition, implying that acquisition and expansion of VRE may be distinct processes. The community composition, diversity, and temporal rate of change did not differ across patients who did (cases) and did not (controls) acquire VRE during their hospitalization. As expected based on the study design, gut communities of cases had a greater abundance of *Enterococcus* than controls after the time at risk.

Gut communities of all subjects demonstrated a rapid and dramatic change during hospitalization that was time dependent. In this population, antibiotic use was prevalent (Table 2), gut microbial communities were remarkably dynamic (Fig. 5), and admission gut microbiota provided very little information about microbiota after the time at risk. Our model of Jaccard distance over time estimated a mean Jaccard distance of 0.79 between two rectal swabs taken on the same day of admission, implying that only 21% of gut microbiota remain constant with resampling within 24 h. Given the significant correlation between Jaccard distance and time, some of this change is likely due to the disruptive pressures that face gut microbiota upon hospitalization (i.e., antibiotics). However, a large portion of this change may represent stochasticity and noise introduced by variation in sample collection and storage. Other studies have found that gut microbiota change dramatically during hospitalization (17–19), but to our knowledge, ours is the largest study to examine this change, the only study to look at the rate of change, and the only study show that most of the change occurs very early (within 72 h). These results have important implications for the clinical use of gut microbiota for therapy, prediction, and risk stratification. Given the rapid change of gut communities upon hospitalization, a single static 16S analysis of gut microbiota may miss subtle dynamics important for VRE acquisition and is subject to a large amount of noise that may obscure a true biologically meaningful association. Future study of the gut microbiota in VRE acquisition may need to move beyond traditional 16S analysis, which can be time-consuming and miss important species-level information (20, 21). Real-time metagenomics and rapid, ultrasensitive quantification technologies hold promise as tools with better resolution to evaluate these processes (22, 23).

Despite the dramatic change in community structure, we did find some evidence of colonization resistance, as admission microbiota were predictive of *Enterococcus* abundance at the time of VRE detection. VRE-colonized subjects with *Blautia* had less *Enterococcus* expansion, consistent with prior studies (8, 15, 16). We hypothesize that there may be a distinction between VRE acquisition and VRE expansion. In conjunction with earlier studies (8, 16, 24), our findings suggest that commensal anaerobes may play a significant role in suppressing VRE once colonized. In this context, our results further support the possibility of microbiome manipulation to reduce VRE burden even in patients already colonized to prevent progression to VRE infection in the individual patient (15, 24) or transmission into the surrounding environment and other hospitalized patients (7, 8, 16).

We noted that more cases received proton pump inhibitor (PPI) therapy prior to initial sampling than controls (Table 2), consistent with prior studies showing that PPI use is a risk factor for VRE colonization (25, 26). Despite this difference in treatment, we found no meaningful difference in the community structure of cases and controls on admission (Fig. 1). This may imply that the increased risk of VRE colonization from PPI therapy is not mediated through changes in gut microbiota, but by elimination of the gut acid barrier to ingested bacteria (27). We believe these findings are hypothesis generating for future studies of the role of PPI therapy in VRE acquisition.

In this retrospective case-control study, we controlled for multiple confounders with our time- and unit-matched design. We used machine learning algorithms robust to multicollinearity and overfitting , and applied permutation heuristics to correct for feature importance bias and decrease our false discovery rate. This study reveals an opportunity for future studies to delineate key differences in pathophysiology between VRE acquisition and domination.

In summary, VRE acquisition and expansion may be two distinct processes, and efforts to manipulate the microbiome to prevent the spread of VRE may be more beneficial in reducing VRE domination in colonized patients than in preventing VRE acquisition in uncolonized patients. Future studies of the role of the gut microbiota in VRE acquisition may need to move beyond single time point 16S analyses and address the role of temporal dynamics and stochasticity of gut microbiota in the acquisition and expansion of VRE.

## MATERIALS AND METHODS

### Study setting and design.

We previously conducted a retrospective case-control study of clinical risk factors for VRE acquisition among patients who did (cases) and did not (controls) acquire VRE during their admissions at the University of Michigan Healthcare System from January 2013 until June 2016 (25). We studied gut microbiome communities in 236 rectal swab samples from 59 matched pairs of case and control subjects from patients admitted to the University of Michigan Hospital in 2016. Sixty-four out of 118 subjects in this study (54%) were a part of our previous clinical risk factor analysis. The remaining subjects were admitted from June to December 2016 (outside the time frame of the previous study by 6 months). The University of Michigan Healthcare system consists of ∼1,000 inpatient beds and serves as a tertiary referral hospital for southeastern Michigan. The institutional review board at the University of Michigan approved the study before its initiation.

### VRE case definition.

The infection control practice throughout the study period was to perform routine surveillance for VRE on eight adult units, including intensive care units, the hematology and oncology ward, and the bone marrow transplant ward. All patients were routinely screened on admission and weekly thereafter with rectal swabs that were tested by Bio-Rad VRESelect chromogenic medium to detect VRE. Cases were defined as subjects with an initial negative swab followed by a positive swab when evaluated by this selective culture. We further identified the “time at risk” for each case patient, here defined as the time elapsed between admission and positive VRE screen. We matched each case subject to a control subject with an initial negative swab followed by repeat negative swab within the same time at risk (±5%). An additional matching factor was the unit from which the first positive VRE was recovered for cases or the matched swab after the time at risk for controls.

### Bacterial DNA isolation.

Genomic DNA was extracted from rectal swabs resuspended in 360 μl ATL buffer (Qiagen DNeasy blood and tissue kit) and homogenized in fecal DNA bead tubes using a modified protocol previously demonstrated to isolate bacterial DNA (28, 29). Sterile laboratory water and AE buffer (10 mM Tris-Cl, 0.5 mM EDTA; pH 9.0) used in DNA isolation were collected and analyzed as potential sources of contamination. ZymoBIOMICS microbial community DNA standard (Zymo Research catalog no. D6306) was sequenced for error analysis.

### 16S rRNA gene sequencing.

The V4 region of the 16S rRNA gene was amplified using published primers and the dual-indexing sequencing strategy developed previously (28). Sequencing was performed using the Illumina MiSeq platform (San Diego, CA) and a MiSeq reagent kit V2 (500 cycles) according to the manufacturer’s instructions with modifications found in the standard operating procedure of the laboratory of Patrick Schloss (28, 30). Accuprime high-fidelity *Taq* was used in place of Accuprime Pfx SuperMix (31). Primary PCR cycling conditions were 95°C for 2 min, followed by 20 cycles of touchdown PCR (1 cycle consisting of 95°C for 20 s, 60°C for 20 s and decreasing 0.3 degrees each cycle, 72°C for 5 min), and then 20 cycles of standard PCR (1 cycle consisting of 95°C for 20 s, 55°C for 15 s, and 72°C for 5 min), and finished with 72°C for 10 min.

### Statistical analyses.

Sequence data were processed and analyzed using the software mothur v.1.43.0 (32) according to the standard operating procedure for MiSeq sequence data using a minimum sequence length of 250 bp (28, 33). To summarize, the SILVA rRNA database (34) was used as a reference for sequence alignment and taxonomic classification. K-mer searching with 8-mers was used to assign raw sequences to their closest matching template in the reference database, and pairwise alignment was performed with the Needleman-Wunsch (35) and NAST algorithms (36). A k-mer-based naive Bayesian classifier (37) was used to assign sequences to their correct taxonomy with a bootstrap confidence score threshold of 80. Pairwise distances between aligned sequences were calculated by the method employed by Sogin et al. (38), where pairwise distance equals mismatches, including indels, divided by sequence length. A distance matrix was passed to the OptiCLUST clustering algorithm (39) to cluster sequences into “operational taxonomic units” (OTUs) by maximizing the Matthews correlation coefficient with a dissimilarity threshold of 3% (40).

After clustering and classification of raw sequencing data, we evaluated differences in community structure with permutational multivariate analysis of variance (PERMANOVA) in the *vegan* package (v 2.0-4) (41) in R (v 3.6.4) (42). We performed resampling of multiple generalized linear models with the *mvabund* (43) package in R to look for individual OTU differences between communities. We set a significance threshold of 0.01 after adjusting for multiple comparisons using a stepdown resampling procedure to reduce the type I error rate (44). We confirmed individual OTU differences with random forest classification and regression models built with the *ranger* package in R (v 0.11.2) (45). We used the *caret* (v 6.0-84) (46) package in R for cross-validation and to optimize the hyperparameters of the number of decision trees in the model and the number of features considered by each tree when splitting a node. We corrected for feature importance bias in random forest models with a permutation importance (PIMP) heuristic developed by Altmann et al. (47). All OTUs were included in diversity and abundance analyses. We performed bivariate analysis with conditional logistic regression using the *survival* (v 3.1-8) package in R (48, 49). Differences in means of final *Enterococcus* abundance were compared with the nonparametric Mann-Whitney U test. We used the *vegan* package in R to calculate both the average species diversity in an individual rectal swab (Shannon diversity) and the change in community structure between the initial swab and second swab for each subject (Jaccard distance). We used Spearman’s rank correlation coefficient to determine whether Jaccard distance was significantly correlated with the time between swabs, and we used nonlinear least-squares regression to fit a model of Jaccard distance over time for cases and controls.

### Adequacy of sequencing.

We performed 16S rRNA gene amplicon sequencing on 236 rectal swab specimens and 15 negative-control specimens, which identified 1,188 unique operational taxonomic units (genus-level bacterial taxa) at a dissimilarity threshold of 3%. After bioinformatics processing, the mean number of reads per sample was 71,484 ± 2,684. No specimens were excluded from the analysis. Rectal swab specimens had clear differences in community structure compared to control specimens, which was confirmed as statistically significant using multiple methods of hypothesis testing (mvabund and PERMANOVA [adonis], *P* < 0.01 for both) (see Fig. S1 in the supplemental material). Sequences generated from negative-control specimens were dominated by a single *Pseudomonas*-classified OTU (OTU001). This OTU was included in all reported analyses, though the exclusion of this OTU did not affect any of the reported results.

10.1128/mSphere.00537-20.3 FIG S1 Comparison of rectal swab communities with negative sequencing controls. A total of 236 rectal swab specimens and 13 negative-control specimens were studied using 16S rRNA gene amplicon sequencing. Bacterial communities in rectal swabs were distinct from those detected in negative controls, both visually and via permutation testing (*P* < 0.001 by PERMANOVA). Download FIG S1, EPS file, 1.6 MB. Copyright © 2020 Chanderraj et al. 2020 Chanderraj et al. This content is distributed under the terms of the Creative Commons Attribution 4.0 International license.

10.1128/mSphere.00537-20.4 FIG S2 Random forest identifies OTUs on admission is predictive of final *Enterococcus* abundance. We built a random forest regression model using admission microbiota and antimicrobial exposures to predict the abundance of *Enterococcus* at the time of VRE acquisition. Seven individual OTUs were significant features at *P* < 0.01 after correcting for feature importance bias. Download FIG S2, EPS file, 1.4 MB. Copyright © 2020 Chanderraj et al. 2020 Chanderraj et al. This content is distributed under the terms of the Creative Commons Attribution 4.0 International license.

### Data availability.

Sequences are available via the NCBI Sequence Read Archive (accession number PRJNA633879). OTU tables, taxonomy classification tables, and metadata tables are available at https://github.com/rishichanderraj/Microbiota_Predictors_VRE_Acquisition. We have excluded protected health information (PHI) attached with this metadata. Potential collaborators are welcome to contact our group with reasonable requests that guarantee patient safety and privacy.
