# Supplementary material for: Gut Microbiota Predict Enterococcus Expansion but Not Vancomycin-Resistant Enterococcus Acquisition
Source: mSphere. 2020 Nov 18;5(6):e00537-20. doi: 10.1128/mSphere.00537-20 (PMC7677005; doi:10.1128/mSphere.00537-20)
Supplement: TABLE S1 [file mSphere.00537-20-st001.docx]

**Supplemental Material:**

| **Table 1. Abundance of Enterococcus after time at risk stratified by presence of individual bacteria on admission swab** | | | |  |
| --- | --- | --- | --- | --- |
|  | *Absent*  Mean relative abundance (n) | *Present*  Mean relative abundance (n) | 95% CI difference in means  (lower bound, upper bound) | P-value |
| Otu0092-Blautia | 23.25 (49) | 4.61 (10) | (-0.77, -21.52) | ***0.007 ***** |
| Otu0026-Lactobacillus | 13.42 (40) | 31.64 (19) | (3.68, 28.83) | ***0.004 ***** |
| Otu0002-Enterobactereciae | 29.52 (27) | 13.2 (16) | (-0.16, -21.61) | 0.038 |
| Otu0019-Phascolarctobacterium | 21.83 (43) | 13.97 (16) | (-13.53, 1.93) | 0.286 |
| Otu0039-Prevotella | 17.38 (53) | 36.78 (6) | (0.94, 46.11) | 0.045 |
| Otu0040-Prevotellaceae | 20.36 (56) | 36.78 (3) | (-39.28, 0.65) | 0.194 |
| Otu0043-Bifidobacterium | 21.3 (47) | 12.79 (12) | (-13.68, 2.21) | 0.195 |
|  | | | |  |
