# Supplementary material for: Gut Microbiota Predict Enterococcus Expansion but Not Vancomycin-Resistant Enterococcus Acquisition
Source: mSphere. 2020 Nov 18;5(6):e00537-20. doi: 10.1128/mSphere.00537-20 (PMC7677005; doi:10.1128/mSphere.00537-20)
Supplement: TABLE S2 [file mSphere.00537-20-st002.docx]

**Supplemental Material:**

| **Table 2. Abundance of Enterococcus after time at risk stratified by presence of individual bacteria on admission swab *for controls*** | | |  |
| --- | --- | --- | --- |
|  | *Absent*  Mean relative abundance (n) | *Present*  Mean relative abundance (n) | P-value |
| Otu0092-Blautia | 4.05 (20) | 0.32 (39) | ***2.4*10^-8^ ****** |
| Otu0026-Lactobacillus | 0.38 (41) | 3.99 (18) | ***0.003***** |
| Otu0002-Enterobactereciae | 1.88 (35) | 2.50 (24) | **2.3*10^-4^ ***** |
| Otu0019-Phascolarctobacterium | 4.04 (56) | 0.33 (3) | **4.3*10^-6^ ***** |
| Otu0039-Prevotella | 4.13 (48) | 0.25 (11) | **1.1*10^-13^ ***** |
| Otu0040-Prevotellaceae | 4.24 (34) | 0.13 (25) | **2.3*10^-13^ ***** |
| Otu0043-Bifidobacterium | 3.74 (37) | 0.63 (22) | **7.2*10^-5^ ***** |
|  | | |  |
